# A bivalent protein r-PB, comprising PA and BclA immunodominant regions for comprehensive protection against *Bacillus anthracis*

**DOI:** 10.1038/s41598-018-25502-9

**Published:** 2018-05-08

**Authors:** Saugata Majumder, Shreya Das, Vikas Somani, Shivakiran S. Makam, Kingston J. Joseph, Rakesh Bhatnagar

**Affiliations:** 1 Microbiology Division, Defence Food Research Laboratory, Defence Research Development Organisation, Mysore, 570011 India; 2 School of Biotechnology, Jawaharlal Nehru University, New Delhi, 110067 India

## Abstract

Anthrax infection is primarily initiated by *B. anthracis* endospores that on entry into the host germinate to vegetative cells and cause severe bacteremia and toxaemia employing an array of host colonisation factors and the lethal tripartite toxin. The protective efficacy of conventional protective antigen (PA) based anthrax vaccines is improved by co-administration with inactivated spores or its components. In the present study, using structural vaccinology rationale we synthesized a bivalent protein r-PB encompassing toxin (PAIV) and spore components (BclACTD) and characterized its protective efficacy against *B. anthracis* infection. Active immunization of mice with r-PB generated high titer circulating antibodies which facilitated the phagocytic uptake of spores, inhibited their germination to vegetative cells and completely neutralized anthrax toxins *in vivo* resulting in 100 % survival against anthrax toxin challenge. Proliferation of CD4+ T cell subsets with up-regulation of Th1 (IFN-γ, IL-2, and IL-12), Th2 (IL-5, IL-10) cytokines and balanced expression of IgG1:IgG2a antibody isotypes indicated the stimulation of both Th1 and Th2 subsets. The immunized mice exhibited 100 % survival upon challenge with *B. anthracis* spores or toxin indicating the ability of r-PB to provide comprehensive protection against anthrax. Our results thus demonstrate r-PB an efficient vaccine candidate against anthrax infection.

## Introduction

*Bacillus anthracis* the causative agent of anthrax is a Gram positive, spore forming bacilli that causes bacteraemia and toxemia in its systemic form through an array of virulence factors^1^. The spores persist in environment for decades and are the natural source of infection when they enter the host through open wounds, orogastric route or through inhalation; the latter being most fatal among all the modes of infection^2,3^. Further, injectional anthrax mediated hypothetically by heroin contaminated with *B. anthracis* spores is an emerging form of infection observed among intravenous drug users^1^. During the early stages of infection, endospores phagocytosed by macrophages retain their viability, germinates and multiplies by manipulating the host immune cell establishing a brief intra-cellular existence before lysing the macrophages to enter the host tissue as vegetative forms^4^. These vegetative forms of the bacteria evade host phagocytosis owing to their poly-γ-D-glutamic acid capsule and produce the tripartite toxin components, protective antigen (PA): the common B subunit and the two alternative A subunits edema factor (EF) and lethal factor (LF). The EF and LF combine with PA individually to form AB type cytotoxins namely edema toxin (ETx) and lethal toxin (LeTx) respectively^5^. LeTx results in macrophage and host cell death while ETx is implicated in phagocyte inhibition and massive edema encountered during anthrax infection^6^.

Vaccination serves as an effective pre-exposure prophylactic measure against anthrax. Presently, Anthrax Vaccine Adsorbed (AVA, Biothrax) licensed in UK and Anthrax Vaccine Precipitate (AVP) licensed in USA are the vaccines available for human use. AVA and AVP are prepared from the cell free culture supernatant of attenuated, *B. anthracis* strains V770-NP1-R and Sterne 34 F2 respectively followed by adsorption to aluminium hydroxide gel (AVA) or precipitate of potassium aluminium sulphate (AVP) respectively^7,8^. The receptor binding protein PA serves as the predominant protective component in these acellular vaccines^9^. In spite of their ability to provide significant levels of protection, these vaccines have an ill-defined general composition, may theoretically cause residual toxicity due to co-adsorption of EF and LF along with PA resulting in lymphadenopathy; disorders in immune system, requires multiple doses, have lot-to-lot variation, limited shelf life and are therefore not recommended to certain population such as pregnant women or people under 18 years of age^10,11^. Alternatively, non-encapsulated, toxigenic, *B. anthracis* Sterne strain licensed for veterinary usage are not used in humans due to troublesome variations in virulence leading to occasional death of immunized animals and declining potency^12^ thereby, necessitating the development of a more optimal anthrax prophylactic.

The *B. anthracis* protective antigen (PA) which plays a central role in anthrax infection consists of four folding domains: an amino terminal domain (domain 1, residues 1–258) enabling proteolytic activation of PA, two heptamerization domains (domain 2-residues 259–487; domain 3-residues 488–595) implicated in membrane insertion and translocation of EF and LF into the host cytosol and a carboxy terminal receptor binding domain (domain 4, residues 596–795)^13^. Mutations between 679–693 amino acids in domain IV hinders the binding of PA83 to host cell receptor and the subsequent internalization of EF and LF thereby, inhibiting toxin formation^14^ while the B-cell epitopes residing in this domain generate high titer neutralizing antibodies and provides protective immunity equivalent to that achieved using entire PA in vaccine design^15–17^. The protection provided by the PA based vaccines is further augmented by spore antigens^1^.

Exosporium components that form the outermost surface of *B. anthracis* spore are the first point of interaction with the host immune system during initial stages of infection^18^ and have been evaluated for their ability to augment the protective efficacy of PA based vaccines^1,19,20^. The *Bacillus* collagen like antigen (BclA) is the major glycoprotein found on the exosporium appendages and contains three domains: a 38 amino acid N terminal domain (NTD) apparently processed to 19 amino acids, a central collagen like region with Xaa-Yaa-Gly (XXG) repeats and a 134 amino acid C terminal domain (CTD). This immunodominant glycoprotein is particularly a prominent target in *B. anthracis* spore vaccine design as the antibody response generated against spores is directed primarily against the protein component of BclA and not its carbohydrate constituents^21^ and immunization with BclA-encoding plasmids could generate protective immunity against *B. anthracis* spores in murine model^12,22^.

The immense potential of structural biology in designing and developing effective vaccines has led to the emergence of a new branch of science; structural vaccinology, which aims at identification of protective domains in the immunogenic proteins of a particular pathogen or multiple pathogens to rationally design and construct multivalent protein comprising two or more such domains^23,24^. Therefore, in the present study, following structural vaccinology rationale, an attempt was made to generate a bivalent sub unit vaccine candidate r-PB encompassing the domain IV of PA and C terminal domain (CTD) of BclA and further characterize its ability to generate protective immunity against *B. anthracis* infection in murine model.

## Results

### Prokaryotic expression and characterization of r-PB

**Figure 1:**
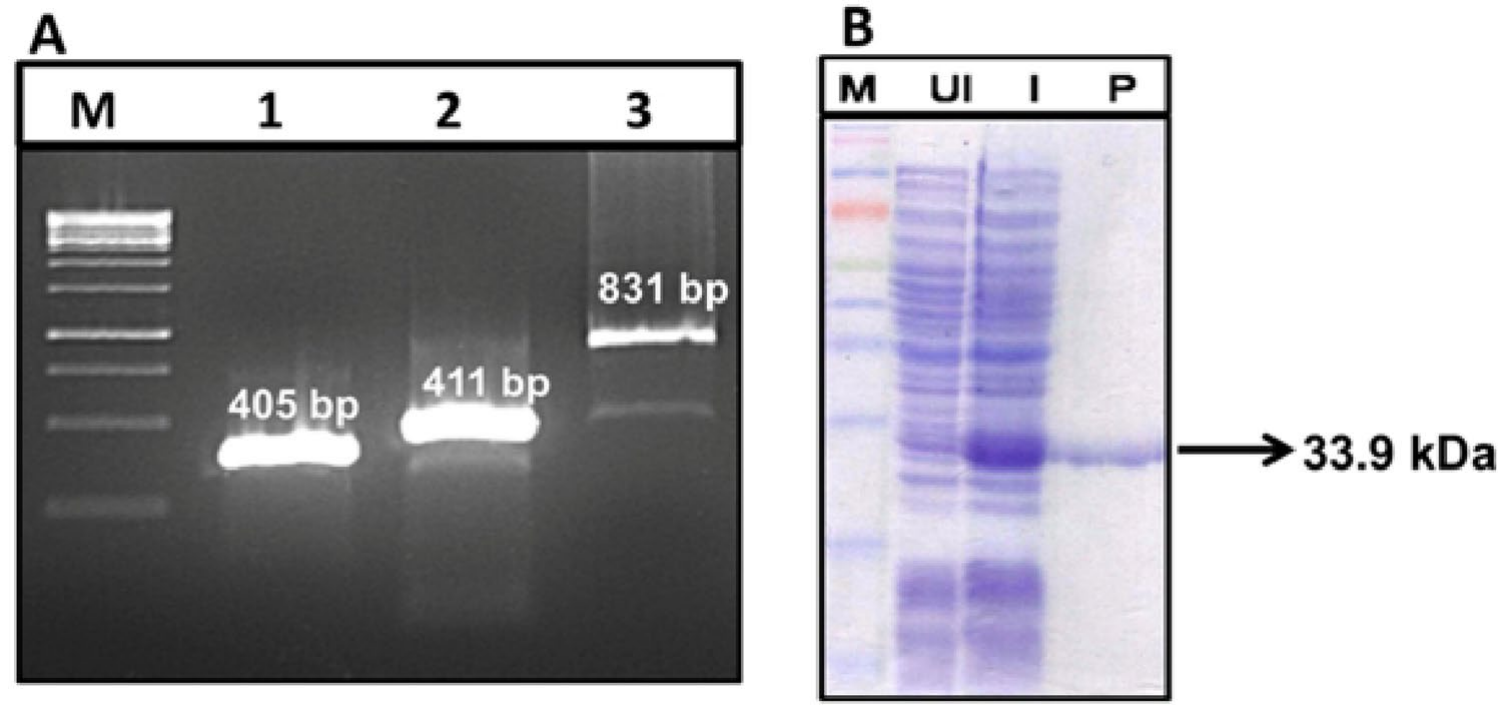
Construction and expression of r-PB. (**A**) Agarose gel electrophoresis of nucleotide fragments; *bclA*1 (bclACTD) (Lane 1); *pag* (Domain IV) (Lane 2); *PB* (Lane 3). (**B**) SDS-PAGE of whole cell lysate from un-induced and IPTG induced r-PB recombinant *E.coli* clones stained with Coomassie Blue: UI: Un-induced clone, I: Induced Clone, P: r-PB purified protein.

The bivalent fusion gene *PB*, encompassing domain IV of PA (PAIV) and C-terminal binding region of BclA (BclACTD) was designed using structural vaccinology approach and synthesized by splicing 411 bp of *pag* and 405 bp of *bclA* genes through the flexible G4S spacer (15 bp) by SOE-PCR (Supplementary File 1). The expression of r-PB (33.9 kDa) in *E. coli* BL21 (DE3) host was observed in  SDS-PAGE gel stained with Coomassie blue (Fig. 1A). A total of 20 mg r-PB per gram of bacterial pellet was obtained after purification by immobilized metal affinity chromatography. The LPS content in both r-PB and r-PA protein preparations was below 0.6 EU/ml as determined by the *Limulus* Amoebocyte Lysate (LAL) assay kit (Lonza, India) and was within the prescribed limits (<20 EU/ml) against recombinant proteins^25^.

### Immunogenicity of r-PB and r-PA

**Figure 2:**
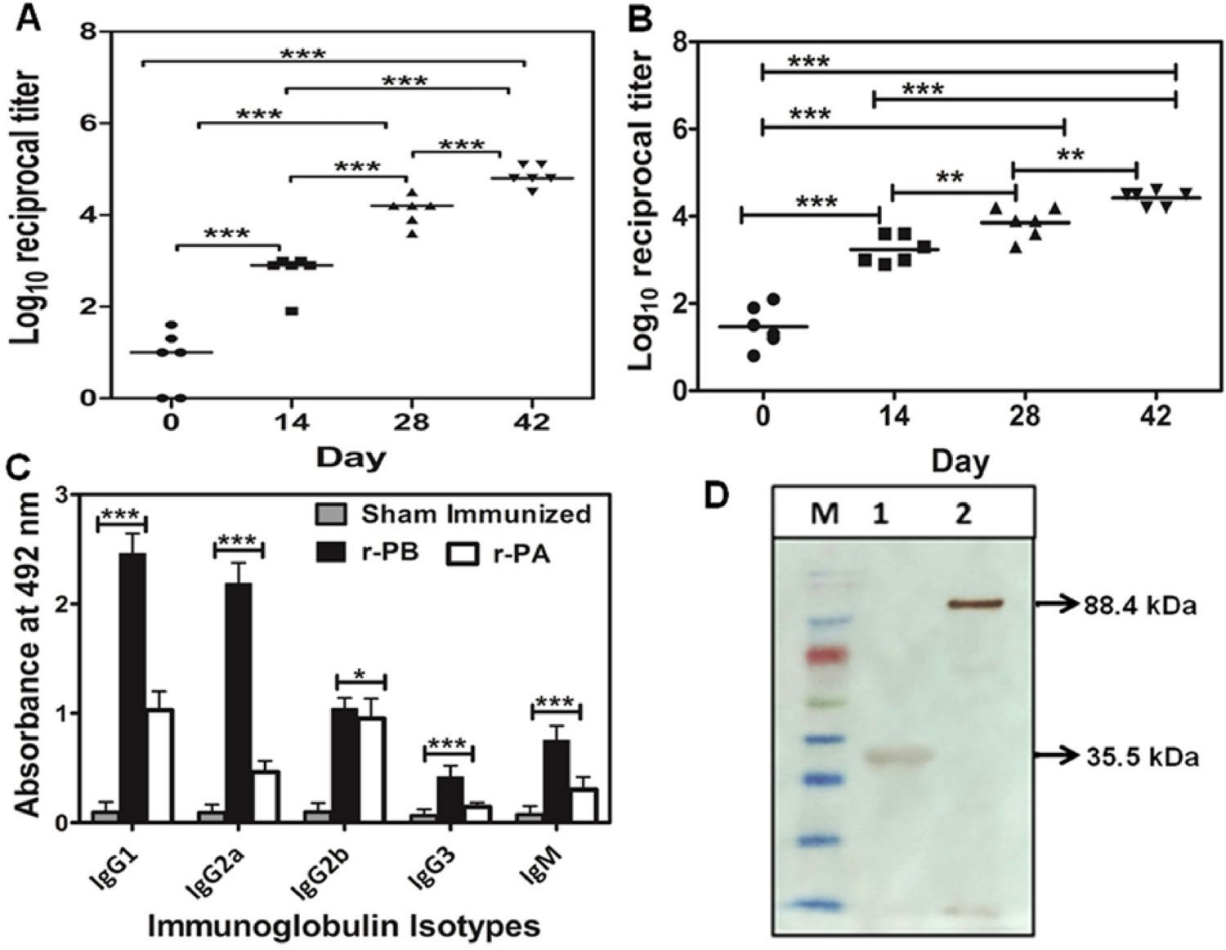
Characterization of r-PB antibody. (**A**) Anti r-PB and (**B**) anti-r-PA specific end point titres of sera collected on 0^th^, 14^th^, 28^th^ and 42^nd^ day of immunization schedule from 6 randomly selected r-PB or r-PA immunized mice respectively were determined by indirect ELISA and plotted. (**C**) Determination of r-PB and anti-r-PA specific antibody subclasses on the 42^nd^ day (1:1000^th^ dilution) sera. The experiments were performed in triplicates and the data are represented in mean ± S.D. (**D**) Western blot analysis to show the reactivity of anti-r-PB sera with BclA (Lane 1) extracted from spores and PA from *B. anthracis* culture supernatant (Lane 2).

A substantial and progressive induction of anti-r-PA and anti r-PB antibody was observed in the sera of s.c. (subcutaneous) immunized BALB/c mice. While primary immunization elicited end point titres of 1:4000 (log 3.602 ± 0.301) for both the proteins, after 42 days of the immunization schedule the anti-r-PB titres increased upto 1:64,000 (log 4.806 ± 0.279) (Fig. 2A) while the anti-r-PA titres were 1:32,000 (log 4.505 ± 0.109) (Fig. 2B). Isotyping analysis of the anti-r-PB antibodies revealed predominance of IgG1 and IgG2a antibody isotypes with IgG1:IgG2a ratio of 1.12 indicating a balanced Th1 and Th2 immune response for anti-r-PB. Alternatively, the anti-r-PA specific IgG1:IgG2a ratio was 2.04 indicating a Th2 biased immune response. Immunization with the proteins generated IgM titres reflecting the elicitation of innate immune response (Fig. 2C). Further anti-r-PB hyperimmune serum reacted specifically with PA from *B. anthracis* culture supernatant and BclA from *B. anthracis* spore suspension generating lucid bands at 88.4 kDa and 35.5 kDa respectively (Fig. 2D).

### Opsonophagocytic Assay

**Figure 3:**
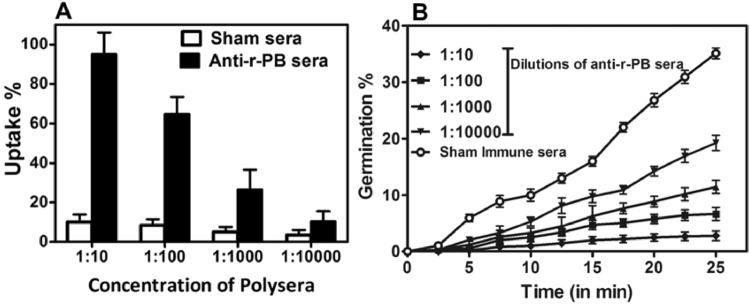
Opsonophagocytosis and spore germination inhibition assay. Enhanced opsonophagocytosis of *B. anthracis* BA10 spores by macrophage cell lines (Raw 264.7) and *in vitro* inhibition of germination was observed when spores were incubated with anti-r-PB antibodies. (**A**) Uptake of spores pre-treated with ten-fold serial dilutions of anti-r-PB antibodies or sham sera by Raw 264.7 macrophage cells after 45 min incubation. (**B**) Germination of spores pre-treated as described above in germination media at intervals of 2.5 min till 25 min of incubation. The data in (**A**) and (**B**) were obtained from two independent experiments, each performed in triplicates and the data is represented in mean ± S.D.

The ability of anti-r-PB antibodies to promote phagocytosis was evaluated by enumerating the bacterial cells released from RAW 264.7 macrophage cells that were exposed to anti-r-PB opsonised spores. Maximum uptake (95.1 %) of *B. anthracis* spores was observed with 1:10 dilution of anti-r-PB sera while the spore uptake gradually declined with higher antibody dilutions. The percentage spore uptake ranged from 95.1 % to 10.5 % for 1:10 to 1:10,000 dilutions respectively. Inconspicuous increase in *B. anthracis* spore uptake was observed in case of macrophage cells incubated with sham sera dilutions (Fig. 3A).

### Inhibition of spore germination by anti-r-PB sera

Spore, being the infective form of *B. anthracis*, the spore germination inhibition capability of the anti-r-PB antibodies was evaluated in the present study. Heat activated, ungerminated *B. anthracis* spores (3 × 10^8^ spores/ml) pre-treated with sham sera and incubated in germination media resulted in 37 % germination upon 25 min incubation. Alternatively, incubation with anti-r-PB antibodies significantly inhibited the germination of *B. anthracis* spores in comparison to the sham sera. A 1:10 dilution of anti-r-PB sera resulted in 2.75 % spore germination while an increase in germination percentage till 19.25 % was observed with 1: 10,000 dilution indicating the ability of anti-r-PB antibodies to inhibit *B.anthracis* spore germination (Fig. 3B).

### *In vitro* proliferation of lymphocytes

**Figure 4:**
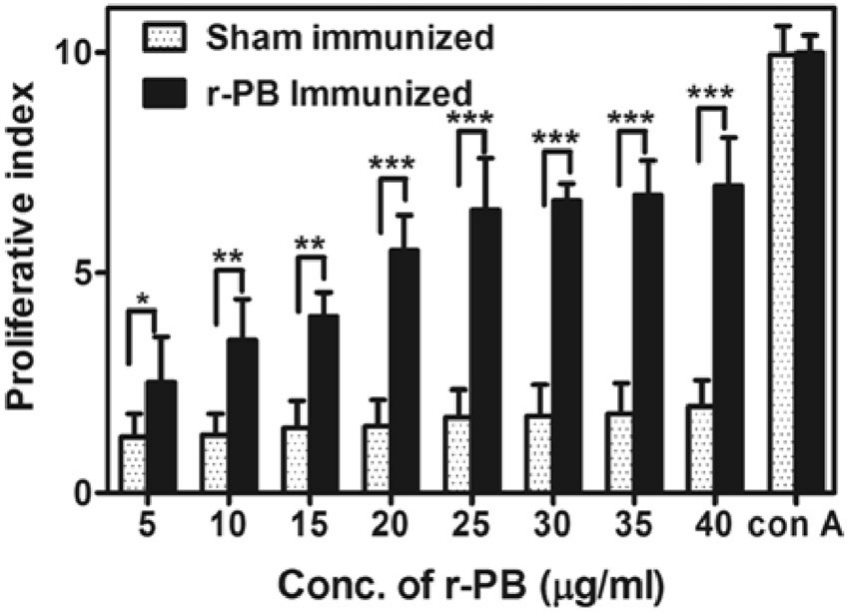
Lymphocyte proliferation assay. Proliferation of r-PB and sham immunized mice splenocytes re-stimulated with gradient concentrations of r-PB determined by MTT assay. Conconavalin A at 20 μg was used as positive control. Experiments were performed in triplicates and data represented as mean PI values ± S.D.

Splenocytes recovered from r-PB and sham immunized mice were evaluated for their ability to proliferate upon exposure to gradient concentration of the antigen (r-PB) with ConA (Conconavalin A) as a positive control. A significant increase (p***) in proliferation (PI = 6.43) was observed upon stimulation with 20 µg/ml r-PB, with no further increase in PI (Proliferative Index) value with increasing concentrations of r-PB. Alternatively no significant increase in proliferation was observed in sham immunized mice splenocytes upon exposure to varying concentrations r-PB antigen. This signifies r-PB as an efficient mitogen capable of stimulating the proliferation of primed T cells (Fig. 4).

### Cytokine Estimation

**Figure 5:**
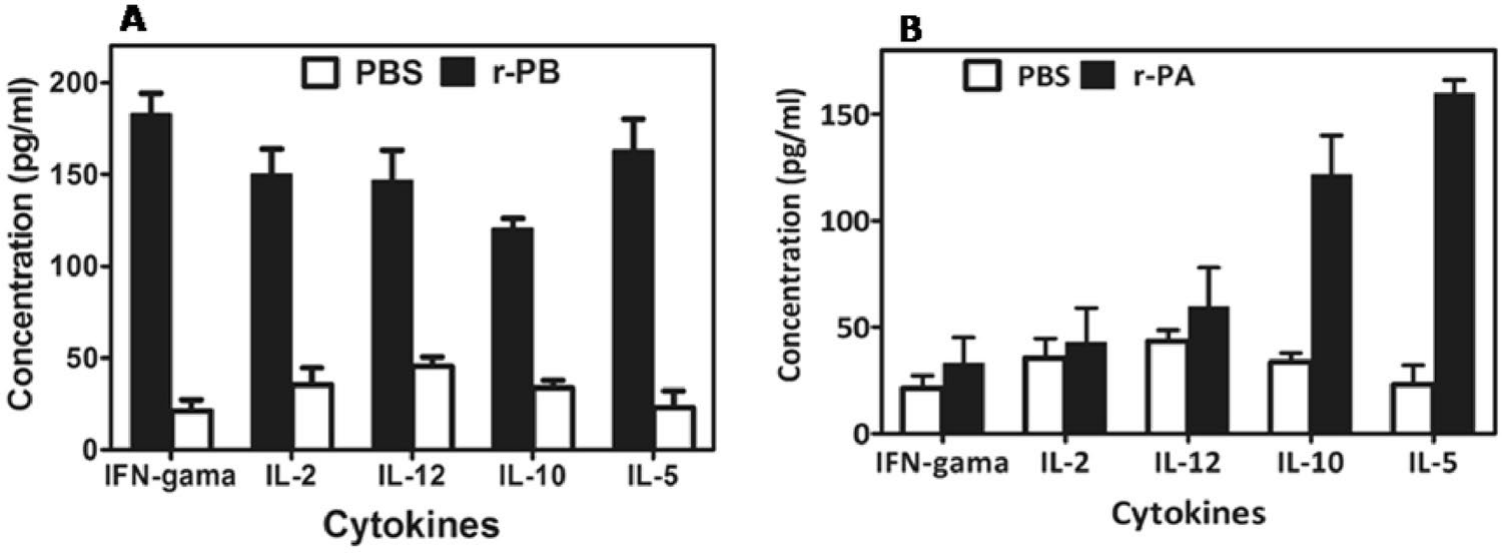
Cytokine profiling in mice immunized with the recombinant proteins r-PB and r-PA. Concentration of Th1 (IFN-γ, IL-2, IL-12) and Th2 (IL-5, L10) cytokines in cell culture supernatants of r-PB or r-PA and sham immunized mice splenocytes individually exposed with (**A**) r-PB or (**B**) r-PA respectively at concentration of 30 µg/ml. The experiments were performed in triplicate and the data represents the mean ± SD of the results determined.

Culture supernatants from r-PB, r-PA and sham immunized mice splenocytes re-stimulated with r-PB or r-PA (30 µg/ml) were evaluated for their ability elicit Th1 (IFN-γ, IL-2, IL-12) and Th2 (IL-5, IL-10) cytokines. The r-PB immunized mice splenocytes exhibited a maximum increase in IFN-γ (8 fold) and IL-5 (7 fold) concentrations followed by IL-2 (4 fold), IL-12 (3 fold) and IL-10 (3 fold) whereas the r-PA immunized mice splenocytes exhibited maximum increase of IL-5 (6.9 fold) and IL-10 (3.5 fold) followed by IL-2 (2.0 fold), IL-12 (1.5 fold) and IFN-γ (1.5 fold) compared to sham immune mice splenocytes. In the r-PB immunized mice, IFN-γ: IL-5 ratio an indirect measure of Th1/Th2 bias was 1.11 thereby indicating a mixed Th1:Th2 response for the r-PB immunized mice whereas in r-PA immunized mice, IL-5: IFN-γ was found to be 4.6 thereby indicating dominant Th2 response. In contrast, the sham immune splenocytes displayed inconspicuous levels of both Th1 and Th2 cytokines (Fig. 5A and B).

### Flow Cytometric Analysis of CD4+ and CD8+ T-cell immune response

**Figure 6:**
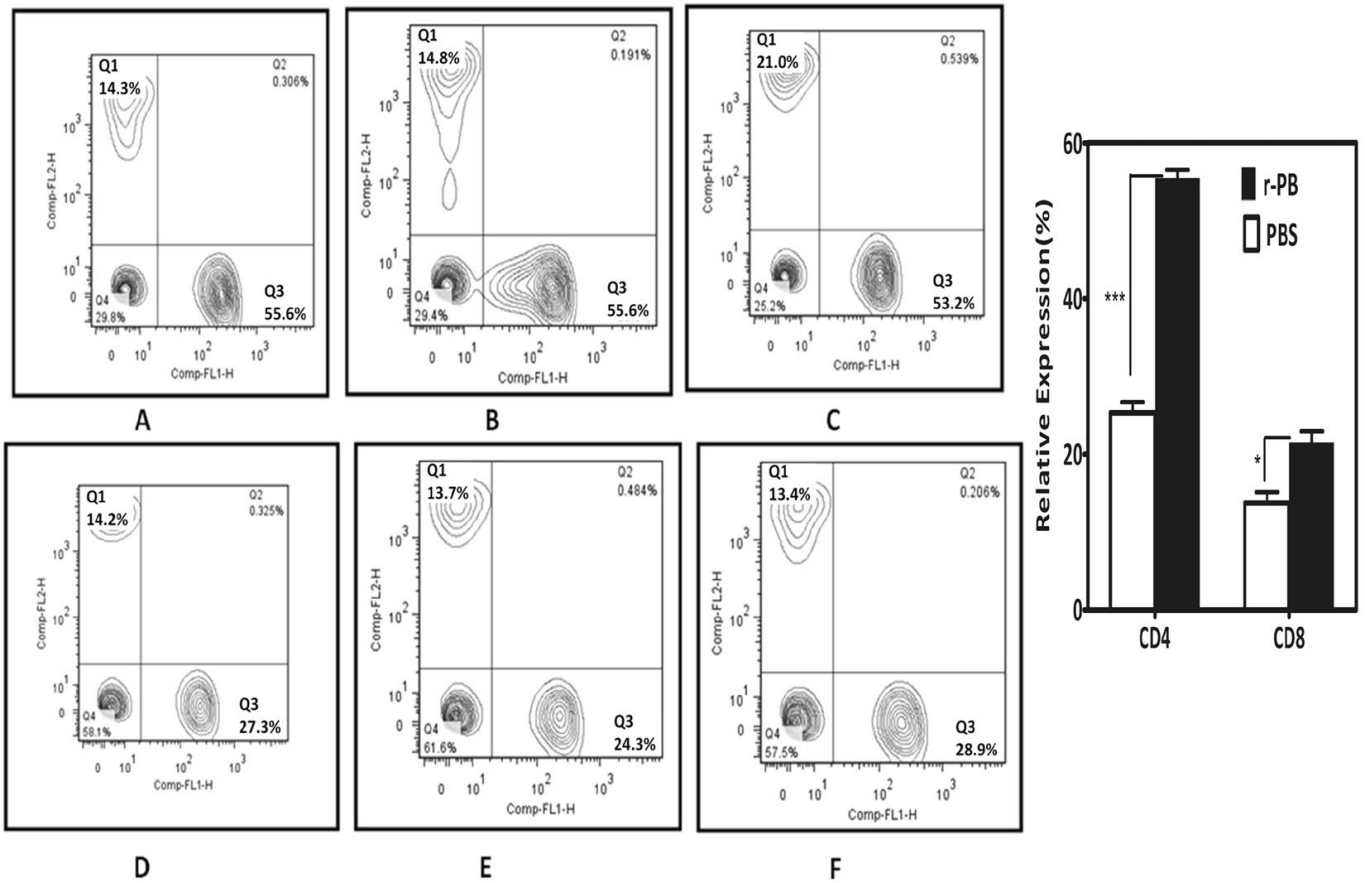
Relative expression of CD4+ and CD8+ T-cells estimated by flow cytometric analysis using FITC anti-mouse CD4 and PE anti-mouse CD8 monoclonal antibodies. Anti-coagulant treated peripheral blood samples from r-PB and sham immunized mice after 42^nd^ day of immunization were used in this assay. (**A**). Representative graphs showing percentage of CD4+ and CD8+ T cells within gated CD3+ T cell population. CD8+ T cell % (Q1), CD4+ and CD8+ T cell % (Q2), CD4+ T cell % (Q3), CD4^−ve^/CD8^−ve^ T cell % (Q4). (**B**) The relative expression percentage of CD4+ and CD8+ cells in r-PB and sham immunized mice.

The peripheral blood samples collected from randomly selected r-PB and sham immunized mice (n = 3 each group) were analysed for the CD4+ and CD8+ T-cells. Though r-PB immunization resulted in the proliferation of both CD4+ and CD8+ T-cell subsets, a relatively higher level of CD4+ T cells (55.4%) proliferation was observed in comparison to the CD8+ T cells (21.53%) (Fig. 6).

### Passive transfer studies

**Figure 7:**
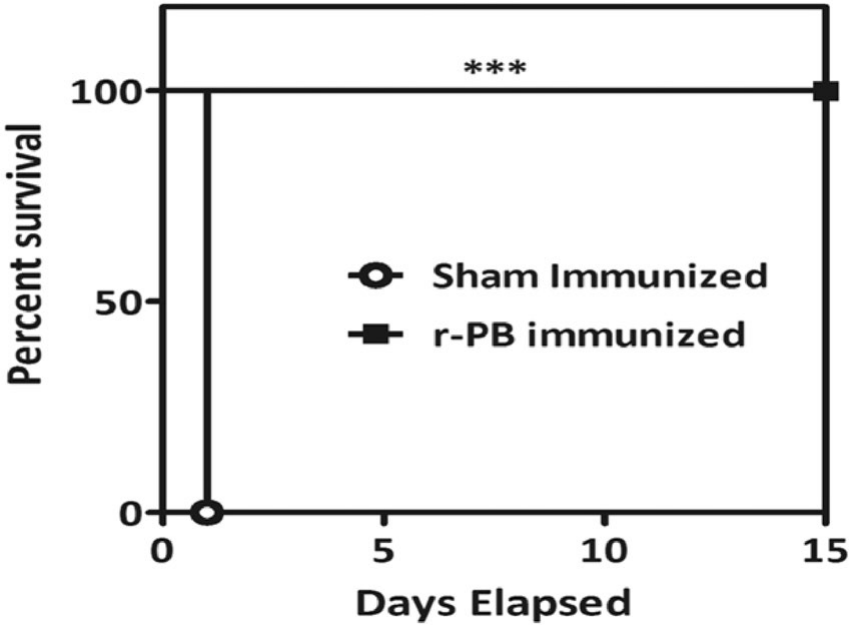
Passive protection of mice against *B. anthracis* crude toxin. Survival percentages of naive female BALB/c mice (n = 6 each group) injected i.p. with concoction of crude anthrax toxin and anti-r-PB over 15 days observation period. Control groups of mice received similar mixtures in polysera from sham immunized mice. Survival percentages were calculated by Kaplan-Meier’s method and was statistically significant (p***).

The anthrax toxin neutralization ability of anti-r-PB antibodies was evaluated by passive transfer studies. The group of animals injected with concoctions of crude anthrax toxins with anti-r-PB antibodies exhibited 100 % survival (6/6) whereas all the animals in control group that received the toxin along with sham sera succumbed to toxin challenge within 24 h (Fig. 7). The difference in survival percentage between r-PB and sham mice was statistically significant (p***). These observations confirmed that anti-r-PB sera can effectively neutralise the crude anthrax toxins.

### Active protection against *B. anthracis* spores and toxin

**Figure 8:**
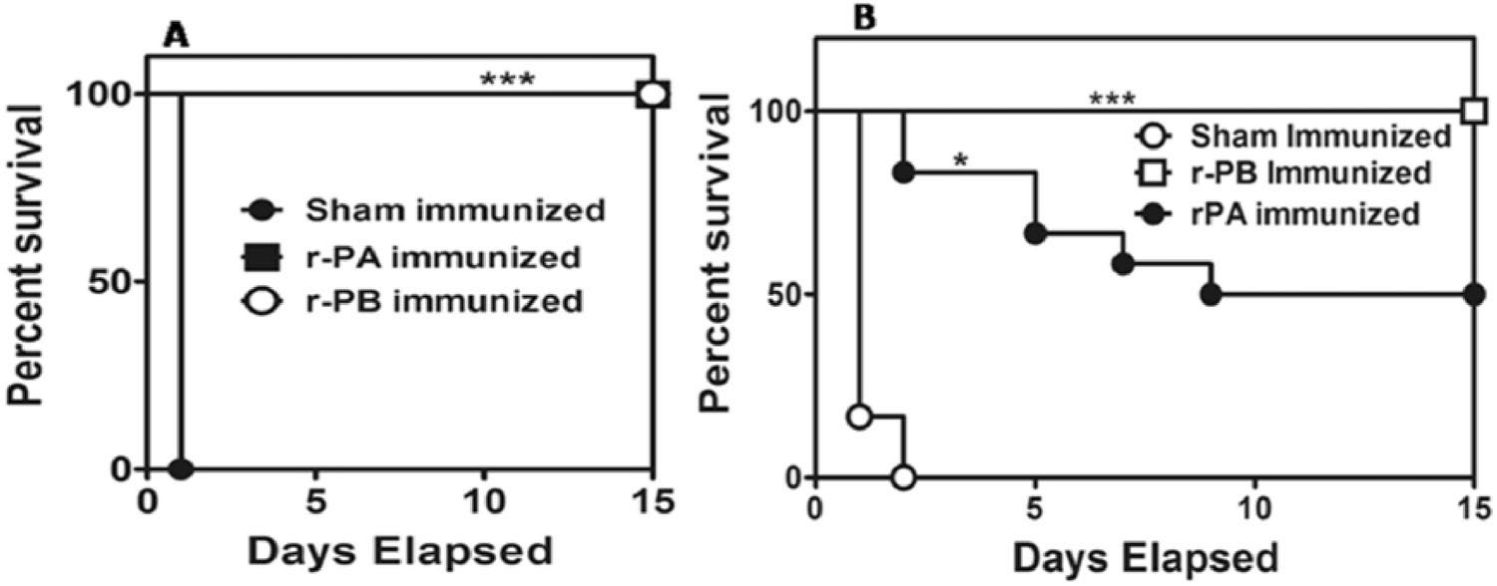
Percentage survival of mice challenged with crude anthrax toxin and spores. Groups of r-PB, r-PA and sham immunized female BALB/c mice (n = 12 each group) were challenged i.p. with 5 × LD_100_ units of crude anthrax toxin (**A**) and 10 × LD_50_ spores (**B**) on the 45^th^ day of immunization schedule and survival was monitored for 15 days. The data in (**A**) and (**B**) were generated from three independent experiments. The protective efficacy of r-PB and r-PA subunit vaccine was calculated by Kaplan-Meier graph to compare percentage survival and was found to be statistically significant (p***).

The protective efficacy of r-PB against anthrax infection was evaluated by challenging the immunized and control mice groups with 5 × LD_100_ doses of crude anthrax toxin or 10 × LD_50_ dose (5 × 10^4^ CFU/ml) of *B. anthracis* Ames strain spores respectively. All the r-PB immunized mice (12/12 in each group) survived both the toxin and spore challenge (Fig. 8A and B) whereas the animals in control groups succumbed to infection with a mean time to death of 24 h after toxin challenge (Fig. 8A) and 48 h after spore challenge (Fig. 8B). Immunization with r-PA resulted in 100 % protection against toxin challenge and 50 % protection against spore challenge till the entire observation period (Fig. 8A and B).

## Discussion

 Majority of the anthrax vaccines primarily rely on the toxin neutralizing antibodies elicited by protective antigen^1^, the host cell binding subunit of anthrax toxins for efficient prophylaxis against *B. anthracis* infection. This antitoxin response though necessary, does not ensure complete protection against anthrax infection in varied instances^1,12,26,27^ underlining the necessity for analogous protective immune response by spore/vegetative cell components to restrict the infection process. The inactivated form of structurally complex and otherwise infectious unit of *B. anthracis* i.e. the spores or its surface components enhances the protective efficacy of PA based subunit vaccines and antitoxin antibodies^1,12^. However, considering the risk of infection associated with inactivated whole spore vaccines^28^ and the possibility for variation in whole spore preparations^29^, a defined spore component that elicits protective immune response can be used to augment the PA protective efficacy. Several recombinant antigens *viz*. rBclA, rExsFA/BxpB and rp5303 from exosporium, the outermost layer of *B. anthracis* spore have been evaluated for their ability to enhance protective efficacy of r-PA based vaccines of which r-BclA showed maximum augmentation of r-PA protective efficacy^1,12^. The BclA C terminal domain (BclACTD) at the distal end of exosporium hair-like naps^30^ possibly binds to the host cell receptors enduring its exposure to the host immune system and could be a plausible choice for vaccine design. Alternatively, the protective efficacy of PA domain IV (PAIV) is well established^31^. Therefore, in the present study r-PB bivalent candidate vaccine was rationally designed and constructed by splicing the PAIV with BclACTD via a G4S linker to have the advantages of comprehensive protection, better immunogenicity devoid of antigenic competition and stearic hindrances otherwise observed in antigen cocktails^32,33^ convenient administration, cost-effective and amenable for stockpiling^34^. Immunization with r-PB generated anti-r-PB antibodies that lucidly reacted with both PA (88.4 kda) and BclA (35.5 kda) from *B. anthracis* toxin and spores in Western blot (Fig. 2D) thereby confirming that epitopic structures of both PAIV and BclACTD domains were maintained in the r-PB bivalent protein.

The r-PB immunized mice upon challenge with *B. anthracis* Ames spores exhibited 100 % survival (Fig. 8B) whereas the survival rate was 50 % in r-PA group (Fig. 8B). Previously, administration of r-PA (PA83) along with spore antigens (BclA, ExsF and p5303) prolonged the time to death (TTD)^1^ while immunization with a DNA vaccine comprising a combination of plasmids encoding PAIV and CLR (Collagen like repeats) deficient version of BclA (BclAD1D3) along with immunomodulators *viz*. mouse interferon-ß promoter stimulator 1 (mIPS-1) and class II MHC trans-activator (CIITA) could confer 90 % protection^12^. To the best of our knowledge this is the first report of achieving 100 % protection against anthrax spore challenge by a bivalent structural vaccine and also demonstrates that the present vaccine design strategy of combining BclA-CTD with the PAIV is appropriate to augment the PA protective efficacy and provide comprehensive protection against *B. anthracis* spores.

It is well established that a concerted induction of both humoral and cell mediated immune responses are required for comprehensive protection against anthrax wherein they act via distinct and independent pathways^35,36^. Therefore, we examined the immunogenicity of r-PB by evaluating the antibody, cytokine responses, proliferation of lymphocytes and cytotoxic/helper T cells elicited by r-PB immunization. Parenteral immunization generated high titer anti-r-PB (1:64000) antibodies that inhibited spore germination *in vitro* (Fig. 3B) and enhanced the phagocytic uptake of *B. anthracis* spores by RAW 264.7 macrophage cells (Fig. 3A). The binding of anti-r-PB antibodies with the spores through their reactivity with BclA on hair like exosporium structures or PA (Fig. 2D) inhibited their germination by hindering the access for germinants at the inoculation site to the spores and also acted as opsonins to facilitate the phagocytic uptake of spores^27^. The extent of spore germination inhibition and opsonophagocytosis observed with anti-r-PB antibodies is similar to that observed with anti-spore^37^, anti-BclA^27^ and anti-PA antibodies^38^ reducing the availability of vegetative forms for progression into systemic stage. In the systemic stage the vegetative forms secrete binary exotoxins ET and LT to bring about the disease etiology. In the present study, the anti-r-PB antibodies completely neutralized native anthrax toxins *in vivo* resulting in 100% survival against anthrax toxin challenge in murine model (Fig. 7). This anthrax toxin neutralizing activity of the anti-r-PB antibodies can be attributed to their binding with PAIV to prevent attachment of PA83 to the cell surface receptor and subsequent heptamerization of PA63^39^. The latter in turn inhibits translocation of EF and LF into host cytosol preventing their cytotoxic effects^17,39^.

During *B. anthracis* infection, LT and ET modulates the host T cell response synergistically by decreasing the production of Th1 cytokines IL-2 and IFN-γ^40^ by the former and blocking movement of T cells^41^, polarising Th2 development against Th1 subset^42^, down regulating the production of IFN-γ and IL12^43^ by the latter. This emphasises the necessity for protective T cell responses along with the adaptive immunity provided by neutralizing antibodies in a rational vaccine design strategy^17,44–46^. Anthrax vaccines, that involve PA or inactivated whole spore mediate protective immunity by induction of CD4+ T cells to elicit elevated levels of Th2 cytokines (IL-5 and IL-10) by the former^47,48^ and Th1 cytokines (IL12 and IFN-γ) by the latter^49^. It can therefore be hypothesized that the incomplete protection provided by these vaccines is probably due to their inability to induce both Th1 and Th2 arms of the immune system. The r-PB induced proliferation of CD4+ T cells (Fig. 6) resulting in the up regulation of both Th1 cytokines (IL-2, IL-12 and IFN-γ) and Th2 cytokines (IL-5 and IL-10) (Fig. 5A). This simultaneous up regulation of both Th1 and Th2 cytokines along with balanced expression of anti-r-PB specific IgG1:IgG2a antibody isotypes in 1:1 ratio indicate the synergistic effect of both Th1 and Th2 subsets attributed to the respective epitopes on BclACTD and PAIV in providing comprehensive protection against anthrax spore and toxin challenge (Fig. 8). The principle Th1 effector cytokine IFN-γ when induced by its precursor IL-12 promotes phagocytosis, chemotaxis, and enables effective killing of *B. anthracis* by macrophages while improving their survival against lethal toxin^49^. Considering this, the r-PB mediated induction of IFN-γ along with elicitation of complement fixing-opsonising antibody isotypes IgG2a (Fig. 2C) can be correlated with the enhanced opsonophagocytosis, inhibition of spore germination (Fig. 3A,B) and PA based complete *B. anthracis* toxin neutralization observed in the present study (Fig. 7). In addition, the deterrence to anti-anthrax adaptive immune responses owing to LT driven IL-2 down regulation could be surmounted by the elevated levels of r-PB induced IL-2 leading to an enhanced T cell proliferation and cellular immunity. Alternatively, the significant rise in levels of the non-complement fixing IgG1 antibody subtype along with the Th2 cytokines IL-5 and IL-10 prevents the uncontrolled Th1 response which would otherwise lead to bacteria induced inflammatory disease and plays crucial role in enhanced B cell survival and antibody production^50^ and this effect was clearly evident from the high anti-r-PB titer (Fig. 2A). However, the anti-inflammatory property of IL-10 skewing Th1 response towards Th2 by suppressing the antigen presenting capacities of APCs^45^ was admirably not observed in the present study.

The immune response to intra-cellular pathogens is mediated primarily by the activation of CD8+ T cells by IL-2 and IFN-γ which in turn elicits protective immunity by lysing the infected cells^51,52^. *B. anthracis* though generally considered as an extra-cellular pathogen, the phagocytised *B. anthracis* spores develop into bacilli and undergo intra-macrophage replication prior to their macrophage mediated transportation to the lymphatics wherein they undergo further multiplication and escape into the host circulatory system for systemic spread and toxin release^53^. Therefore, it is reasonable to hypothesize that the r-PB primed CD8+ T cells could play a role in purging macrophages with intra-cellular spores and bacilli as observed for several other intra-cellular bacterial infections^46,54^ thus contributing to the comprehensive protection by r-PB.

In conclusion, the present study demonstrates the bivalent protein r-PB as an effective vaccine candidate that induces the proliferation of CD4+ T cells with synergistic up-regulation of both Th1 and Th2 cytokines. This simultaneously augments Th1 and Th2 immunity resulting in effective inhibition of spore germination, enhanced opsonophagocytosis and comprehensive protection against both spore and toxin challenge. We also hypothesise a protective role for the r-PB primed CD8+ cells in controlling anthrax infection that has to be established by further studies.

## Materials and Methods

### Bacterial strains, growth conditions and spore preparation

In the present study, pathogenic *Bacillus anthracis* BA10 and *Bacillus anthracis* Ames harbouring both pXO1 and pXO2 plasmids were used. *Escherichia coli* strain BL21DE3 (Thermo Fisher Scientific, India) and SG13009 (Qiagen, Germany) was used for cloning and expression of r-PB and r-PA recombinant proteins respectively. The *B. anthracis* strains and *E. coli* were cultivated in Brain Heart Infusion broth (BHI) and Luria Bertani broth (LB), respectively sourced from Hi-Media, India.

### Toxin extraction and spore preparation

Toxin extraction from *B. anthracis* BA10 was performed as previously described^17^. Briefly, BA10 cells were grown in toxin production media^55^ for 24 h at 37 °C followed by centrifugation at 7800 rpm. The anthrax toxins present in the cell free growth medium were filtered through 0.22 µm filter and precipitated with saturated ammonium sulphate solution followed by dialysis in PBS to remove the excess salts. All the above procedures were performed at 4 °C to minimize toxin degradation. The proteins were further concentrated by lyophilisation and stored at −20 °C for further use. Spores from BA10 and Ames strains were prepared from cultures grown in Modified Germination (G) medium^56^ for 48 h at 37 °C. The culture was heat-treated at 65 °C for 1 h to kill any viable vegetative cell. The spores were washed extensively in cold (4 °C) distilled water to yield a final concentration of 10^8^ colony forming unit (CFU)/ml as determined by plating on BHI plates. Spores were stored in −20 °C for further use.

### Cell lines and media

Raw 264.7 macrophage cell line procured from National Centre for Cell Science, Pune, India were grown in Dulbecco’s Modified Eagle’s medium (DMEM) with 10% FBS, 50 units/ml penicillin, and 50 µg/ml streptomycin. The cells were maintained at 37 °C in 5% CO_2_. All the cell culture media, reagents and chemicals were purchased from Sigma-Aldrich (India), unless mentioned otherwise.

### Ethic Statement

All animal management and research procedures were conducted as per animal use protocols approved by the Institutional Animal Ethical Committee, Defence Food Research Laboratory (DFRL/28/IAEC/CPCSEA), and Jawaharlal Nehru University (JNU) (19/1999/CPCSIA) completely accredited by Committee for the Purpose of Control and Supervision of Experiments on Animals (CPCSEA), India and performed in accordance with their relevant guidelines and regulations. During the course of experiments, mice were maintained in a pathogen free facility and fed with sterile food and water *ad libitum* and all possible efforts were imposed to minimize sufferings.

### Cloning, Expression and Purification of r-PB and r-PA

The fusion gene *r-PB* was constructed by overlap extension PCR (Supplementary File 1) and cloned in *E. coli* BL21 DE3 (Thermo Fisher Scientific, India) as per laboratory manual by Sambrook *et al*., 1989. Briefly, fusion gene *PB* and pRSET A vector (Thermo Fisher Scientific, India) were restricted individually with *KpnI* and *HindIII* and ligated together to form recombinant plasmid r-pRSETA-*PB* that was transformed into chemically competent *E. coli* BL21 DE3. Positive clones were induced with 1 mmol^−1^ Isopropyl b-D-thiogalactopyranoside (Sigma, India), and the r-PB protein expressed was examined on 12 % SDS PAGE. The *E. coli* SG13009 clone harbouring the recombinant plasmid r-pQE30-*pag* that expresses r-PA protein as reported earlier^57^ was used in our study and expression of r-PA protein was induced with 1 mmol^−1^ Isopropyl b-D-thiogalactopyranoside (Sigma, India). The r-PB and r-PA proteins were purified under denaturing conditions by Immobilized Metal Affinity Chromatography using Ni-NTA Agarose column (Qiagen, Bangalore) as per manufacturer’s protocol. The purified proteins were dialysed against PBS + 10 mmol^−1^ arginine (pH 7.4) for 4 h at 4 °C, quantified by Lowry’s method against known BSA standards and stored at −20 °C until further use. The LPS content of the recombinant proteins were determined using the *Limulus* amoebocyte lysate (LAL) test (Lonza, India, Product# F245-06SA) according to manufacturer’s instructions.

### Immunization

Two groups of mice (n = 12 per group) received a primary sub-cutaneous (s.c.) injection of 30 µg of r-PB or r-PA antigen respectively (in 0.1 ml volume) with Freund’s complete adjuvant in 1:1 (v/v) ratio. Subsequently on days 7, 21 and 35 all the animals received boosters with similar concentration of antigen with Freund’s incomplete adjuvant. The control group of animals (n = 12) were sham immunized with 1 × sterile PBS (pH 7.4 ± 0.2) in adjuvant. Blood samples were drawn periodically (days 0, 14, 28, 42) through retro-orbital plexus and the sera was collected and stored in −20 °C for further use.

### Estimation of r-PA and r-PB specific antibody and IgG subclasses

The r-PB or r-PA specific antibody titres were measured using two fold serially diluted sera from 6 randomly selected r-PB or r-PA immunized mice by indirect ELISA. Endpoint titers were determined as maximum antibody dilution when O.D. value was twice more than the mean O.D. value of the control sera. Serum samples collected on 42^nd^ day of immunization (1:1000^th^ dilution) was assayed for r-PB or r-PA specific IgG1, IgG2a, IgG2b, IgG3 and IgM levels using Mouse Antibody Isotyping kit (Sigma, India) as per manufacturer’s instruction.

### Opsonophagocytic and Spore Germination Inhibition Assay

The assays were performed as reported by Welkos *et al*.^38^. Briefly, heat activated, refractile, ungerminated *B. anthracis* BA10 spores (3 × 10^8^ spores/ml) were incubated with tenfold serial dilutions of anti-r-PB antibodies or naive sera in ice for 30 min.

To estimate opsonophagocytosis, antibody treated spores were then added to RAW 264.7 macrophage cells and incubated for 45 min at 37 °C in 5% CO_2_. Infected macrophage cells were washed and incubated with gentamicin to remove any extracellular *B. anthracis* vegetative cells. The macrophages were then washed with cold distilled water and lysed with 100 µ1 of 0.1% Triton X [Sigma, India] and plated on LB agar for enumerating the viable cfu/ml.

To enumerate the germination inhibition, pre-treated spores were added to the germination media (1% (v/v) BHI in water) in 96 well micro-titre wells and incubated for 25 min at 30 °C. Micro-titer wells with spores and germination media were considered as positive control. O.D._560_ was measured at intervals of 2.5 min in micro-titer plate reader (Infinite M200 pro; Tecan, Grodig, Austria). The experiment was performed in triplicates and extent of germination inhibition was analysed by the decline in O.D._560_ and plotted in a graph

### Lymphocyte Proliferation Assay

Splenocytes were harvested^58^ from r-PB and sham immunized mice spleens (n = 6 each group). The splenocytes in DMEM media (5 × 10^5^ cells/well) were primed with r-PB (5–40 µg/well) and incubated at 37 °C for 72 h at 5% CO_2_. ConA at 20 µg /ml concentration was used as positive control. Extracted splenocytes with DMEM media alone were considered as negative control. Proliferation of splenocytes was assessed by incubating with 50 µg of MTT [3-(4, 5-dimethythiazol-2-yl)- 2,5-diphenyl tetrazolium bromide] in dark for 2 h at 37 °C. Reactions were developed with 200 µl of Dimethyl Sulfoxide (DMSO) and O.D. was observed at 570 nm in micro-titer plate reader (Infinite M200 pro; Tecan, Grodig, Austria).

### Cytokine profiling

The Th1 cytokines (IL-2, IL-12, IFN-γ) and Th2 cytokines (IL-5 and IL-10) elicited in r-PB, r-PA and sham immunized groups were estimated by Bio-Plex Pro Mouse Cytokine 8-Plex Immunoassay kit (Bio-Rad, USA) containing fluorescently labelled magnetic beads covalently conjugated with a cocktail of monoclonal antibodies specific for the target cytokines as per manufacturer’s instructions. Briefly, microtiter wells were coated with anti-cytokine antibody coated magnetic beads and incubated for 1 h at 37 °C with equal volumes of culture supernatants from r-PB, r-PA and sham immunized mice splenocytes exposed with r-PB or r-PA (30 µg/ml) respectively. The microtiter wells were washed with Bio-Plex Wash Buffer and probed with biotinylated detection antibody for 1 h at 37 °C. After subsequent washing with Bio-Plex Wash Buffer the wells were incubated with Streptavidin-PE, in dark. The wells were washed and the beads in each well was resuspended with assay buffer and incubated for 30 secs with shaking at 1,100 rpm. Post incubation, the fluorescence was measured on a Bio-Plex 200^®^ array reader and analysed with the Bio-Plex Manager software v3.0.

### Flow Cytometric Analysis of CD4+ and CD8+ T-cell immune response

Flow Cytometry analysis to evaluate CD4+ and CD8+ T cell counts in immunized mice were performed as previously described^59^. Briefly, anti-coagulant treated (EDTA 2 mM) peripheral blood samples obtained from r-PB and sham immunized mice (n = 3 each group) were used for estimation of CD4+ and CD8+ T cell counts. The erythrocytes were lysed with 1:10 diluted lysis solution (FACS lysing solution, BD) for 30 min in dark at 37 °C. After 30 min incubation cells were harvested and washed with Dulbecco’s PBS and 10^6^ cells were stained with FITC anti-mouse CD4+ antibodies (BioLegend, India) and PE anti-mouse CD8+ antibodies (BioLegend, India). A minimum of 20,000 events were counted for each analysis using BD FACS Verse Flow Cytometer (Becton-Dickson, Singapore) and results were analysed using Kaluza software version 3.1 v (Beckman Coulter, USA). The percent activated CD3+ cells were electronic gated among the total lymphocytes obtained and the CD4+ and CD8+ T cell populations in the gated CD3+ T cells were determined.

### Passive transfer studies

Two groups of naïve female BALB/c mice (n = 6 each group) were i.p. immunized with 100 µl of ten-fold diluted pooled anti-r-PB sera and sham sera respectively and challenged with 5 × LD_100_ units of crude anthrax toxin post 24 h of immunization. Mice were observed for signs of weakness and survival was monitored for 15 days. The toxin neutralization efficacy of anti-r-PB was calculated by Kaplan-Meier’s method to compare percentage survival.

### Active protection against *B. anthracis* spores and toxin

Two groups of naive female BALB/c mice (n = 12 each group) were immunized with 30 µg r-PB while two separate groups of mice (n = 12 each group) received r-PA following similar immunization condition. Two control groups each with 12 mice were sham immunized with 1X sterile PBS (pH 7.4 ± 0.2) in adjuvant. On the 45^th^ day of the immunization schedule the r-PB, r-PA and sham immunized mice (n = 12 per group) were challenged with 0.2 ml of 5 × LD_100_ doses of crude anthrax toxin (diluted in sterile PBS, pH 7.4) or 0.1 ml of 10 × LD_50_ dose (5 × 10^4^ CFU/ml) of *B. anthracis* Ames spores in PBS respectively via i.p. route. Mortality and morbidity were recorded for 15 days after post challenge. The above experiment was performed in containment facility (BSL3) at Department of Biotechnology, Jawaharlal Nehru University, Delhi. The protective efficacy of r-PA and r-PB sub unit vaccine against *B. anthracis* spores and toxin was calculated by Kaplan-Meier’s method to compare percentage survival.

### Statistical analysis

The data were represented as mean ± S.D. Mantel-Cox (log rank) test was used to compare the survival curves and Student’s *t*-test was used for other statistical comparisons. All graphical illustrations were constructed. Graph Pad Prism 5 software. Significance (P) value summary: *P ≤ 0.05, **P ≤ 0.001; ***P ≤ 0.0001.

## Electronic supplementary material

Supplementary File 1
