# Supplementary material for: A bivalent protein r-PB, comprising PA and BclA immunodominant regions for comprehensive protection against Bacillus anthracis
Source: Sci Rep. 2018 May 8;8:7242. doi: 10.1038/s41598-018-25502-9 (PMC5940697; doi:10.1038/s41598-018-25502-9)
Supplement: Supplementary file 1 [file 41598_2018_25502_MOESM1_ESM.pdf]

A bivalent protein r-PB, comprising PA and BclA immunodominant regions for comprehensive protection against *Bacillus anthracis*

Saugata Majumder[1], Shreya Das[1], Vikas Somani[2], Shivakiran S. Makam[1], Joseph Kingston J[1]*, Rakesh Bhatnagar[2]

*Corresponding Author

[1]Microbiology Division, Defence Food Research Laboratory, Defence Research Development Organisation, Mysore-570011

[2]School of Biotechnology, Jawaharlal Nehru University, New Delhi 110067

Corresponding Author details:

Email: joseph@dfrl.drdo.in

Ph: +91-821-2579435

Materials and Methods;

Construction of *PB* Chimeric Gene by splicing overlap extension PCR:

DNA was extracted from *B. anthracis* BA10 as per Marmur (1961). The nucleotide sequences of *pag* (Genbank AF306782.1) and *bclA* (Genbank NC_003997.3) were retrieved from NCBI database and primers were custom synthesized from Sigma Aldrich, Bangalore. Nucleotide sequences of PAIV (1930-2340 bp) and BclACTD (742-1146 bp) were spliced via a flexible G4S linker by overlap-extension PCR.

The whole strategy comprised of three important steps as demonstrated in FigS1.

Step1. Amplification of individual fragments with complementary overhangs.

Gene fragments encoding 1930-2340 bp of *pag* and 742-1146 bp of *blcA* PCR were amplified independently by PAF+PAR and bclAF+bclAR primers respectively. In another PCR, glycine linker overhangs were added to modify both the *pag* (PAF+PAGlyR) and *bclA* (bclAF+bclAGlyR) at 3’ and 5’ ends respectively.

Step2. Single Step Fusion PCR

Equimolar ratios of the modified gene fragments were mixed with each other and were spliced together by primer free PCR.

Step3. Nested Amplification of fused gene.

The spliced products were amplified using the extreme primers PACLNF and bclACLNR containing restriction cites (underlined in primer sequences) for *KpnI* and *HindIII* respectively.

PCR Conditions:

Except the PCR in Step2, all other PCRs (MastercyclerPro, Eppendorf, Germany) were kept in 20µl reactionmix containing 50 ng template DNA, 1X pfu PCR buffer (with 2.5 $mmol^{-1}$ MgSO4), 0.2 $mmol^{-1}$ dNTPs mix, 10 $pmol^{-1}$ each primer and 1 unit *pfu* polymerase (Fermentas, New Delhi, India). The PCR conditionswere as follows: initial denaturation at 94°C for4 min, 30 cycles of 1 min denaturation at 94°C, 1 min annealing at 56°C, 1 min extension at 72°C and a finalextension at 72°C for 8 min. The fusion PCR was performed in a 30µl reaction mix containing 100 ng each of purified *pag* and *bclA* fragments (PCR-Clean up kit; Sigma-Aldrich), 1X *pfu* PCR buffer (with 2.5 $mmol^{-1}$ MgSO4), 0.2 $mmol^{-1}$ dNTPs mix and 2 units *pfu* polymerase (Fermentas, New Delhi, India). The PCR conditions for OE-PCR were denaturationat 94°C for 5 min, annealing at 56°C for 1 minand extension at 72 °C for 15 min. All the amplicons were electrophoresed in 1% agarose gel, stained with ethidium-bromide and visualized under UV Transilluminator.

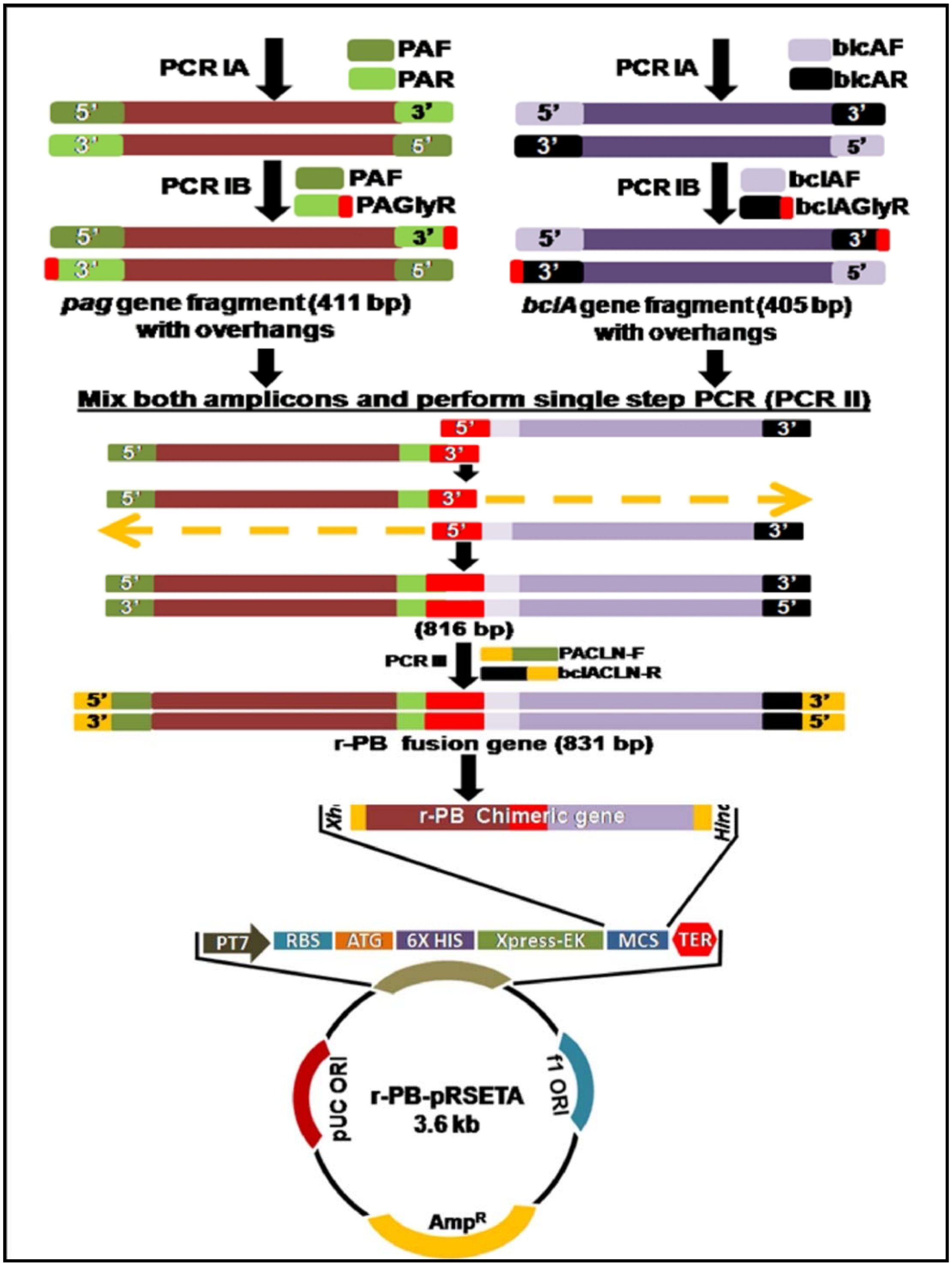

FigS1. Schematic representation of construction of r-PB gene by OE-PCR

Primers for SOE-PCR

Primer	Sequence	No of Bases	Size (bp)
PA-F	GATAGAAATAACATAGCAGTTG	22	411
PA-R	TCCTATCTCATAGCCTTTTTT	21	
bclA-F	GGACTAGGACTTCCAGCAGG	20	405
bclA-R	AGCAACTTTTTCAATAATAATGG	23	
PA-CLN-F	CGGGGTACCGATAGAAATAACATAGCAGTTG	31	831
PA-GLY-R	CTGAACCACCACCACCTCCTATCTCATAGCCTTTTTT	37	
bclAGLY-F	GGTGGTGGTGGTTCAGGACTAGGACTTCCAGCAGG	35	
bclACLN-R	GGGAAGCTTAGCAACTTTTTCAATAATAATGG	32	

Reference:

1. Marmur, J. A procedure for the isolation of deoxyribonucleic acid from micro-organisms. *J Mol Biol*. 3(2): 208IN1-218 (1961).
